# Papillary thyroid carcinoma with nodular fasciitis-like stroma: a rare variant

**DOI:** 10.4322/acr.2021.320

**Published:** 2021-08-20

**Authors:** Mayur Parkhi, Debajyoti Chatterjee, Anand Bardia, Anwin Joseph Kanaval, Ashwani Sood, Uma Nahar Saikia, Naval Bansal

**Affiliations:** 1 Post Graduate Institute and Medical Education and Research, Department of Histopathology, Chandigarh, India; 2 Post Graduate Institute and Medical Education and Research, Department of Nuclear Medicine, Chandigarh, India; 3 Fortis Hospital, Department of Surgery, Mohali, Punjab, India

**Keywords:** Thyroid cancer, papillary, Fasciitis, Myofibroblasts, Beta catenin, Proto-Oncogene Proteins B-raf

## Abstract

Papillary thyroid carcinoma with desmoid-type fibromatosis (PTC-DTF) or nodular fasciitis-like stroma (PTC-NFS) is a rare morphological variant of PTC with a favorable prognosis. There is a paucity of molecular data regarding this entity. We present the case of a 20-year-old female who presented with a palpable mass over the anterior aspect of the neck for the past 3–4 months, which was diagnosed as PTC-NFS. Ultrasonogram of the neck revealed a bulky left lobe of thyroid that contained a well-defined heterogenous lesion measuring around 24 × 26 × 36 mm with involvement of the adjacent isthmus. She underwent a total thyroidectomy with central compartment lymph node dissection. Histological examination revealed a biphasic tumor with epithelial and stromal components resembling nodular fasciitis. Two dissected lymph nodes showed metastasis of the epithelial component only. On immunohistochemistry, *BRAF* mutant protein expression was evident in the epithelial component only, while β-catenin was negative in both the components. The histopathological diagnosis of papillary thyroid carcinoma with nodular fasciitis-like stroma was offered. Sanger sequencing revealed a *BRAF*V600E (c.1799T>A, Val600Glu) mutation. Post-operatively, no residual tumor was detected on ultrasound and radioiodine scans. The patient was doing well at follow-up of 9 months. PTC-NFS/DTF is a histological variant of PTC with a favorable prognosis. Our index case was associated with the *BRAF* mutation, which was restricted to the epithelial component. Thorough sampling of the excised specimen is essential in order not to miss the epithelial component, which, in most reported cases (including ours) appears to be small.

## INTRODUCTION

Papillary thyroid carcinoma with desmoid-type fibromatosis (PTC-DTF)—or nodular fasciitis-like stroma (PTC-NFS)—is a rare morphological variant of PTC, characterized as a biphasic tumor comprising extensive proliferating myofibroblastic stroma and PTC component in varying proportions.^1^ This rare entity has been mentioned under the heading of “PTC with fibromatosis/nodular fasciitis-like stroma” in the recent *WHO Classification of Tumors of the Endocrine Organs* (2017 edition).^1^ Although it is an indolent tumor, its clinical behavior is not well known because there are only a few reported cases. Herein, we describe a 20-year-old young lady who presented with palpable thyroid swelling.

## CASE REPORT

A 20-year-old young female presented with palpable swelling over the anterior aspect of the neck for the past 3–4 months, without any associated pain, dysphagia, or change of voice. On neck examination, the swelling was felt as a firm-to-hard, mobile nodule that was arising from the left lobe of the thyroid. An ultrasonogram (USG) of the neck revealed a bulky left lobe of thyroid that showed a well-defined heterogenous lesion measuring approximately 24 × 26 × 36 mm with involvement of the adjacent isthmus. Mild vascularity within the lesion was seen on color flow imaging. Fine needle aspiration (FNA) confirmed the diagnosis of PTC. She underwent total thyroidectomy with central compartment lymph node dissection in an outside hospital and was referred to our institute for pathology review and further management.

**Figure 1:**
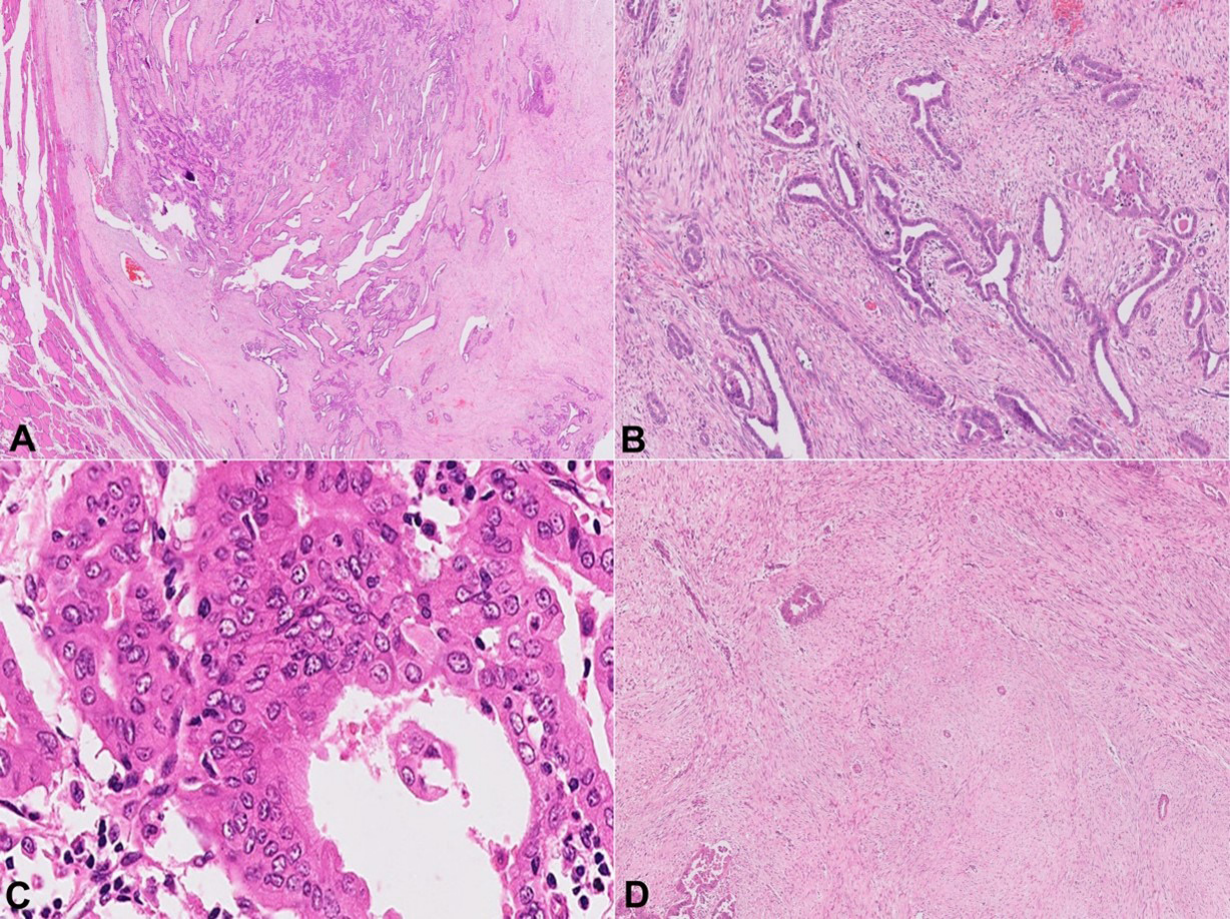
Papillary thyroid carcinoma with nodular fasciitis-like stroma. **A** – A non-encapsulated and well circumscribed tumor showing a biphasic growth pattern with surrounding normal thyroid parenchyma (H&E, 4x); **B** – The epithelial component showing an arrangement in the form of tubulo-papillary and leaf-like or animal-like patterns (H&E, 10x); **C** – The tumor cells are in a single layer with characteristic nuclear chromatin clearing, grooving, and pseudo-inclusions at places (H&E, 40x); **D** – The stroma element shows fascicular arrangement with uniform proliferating benign spindle cells indicating a reactive nature (H&E, 10x).

The pathology review of the total thyroidectomy specimen revealed a well-circumscribed, grayish-white nodule measuring 36 × 35 × 34 mm in the left lobe. The isthmus also showed two tiny grayish-white nodules measuring 3 × 2 mm and 2 × 2 mm. On microscopic examination, a non-encapsulated and well-circumscribed tumor was noted, which showed a biphasic growth pattern. The predominant component was formed by a stromal element (65%–70%) with intervening epithelial component (30%–35%) (Figure 1A). The epithelial component showed tubular and papillary configuration, and at various places, it was compressed by the stromal component in a leaf-like or animal-like configuration (Figure 1B). The epithelial structures were lined by a single layer of moderately pleomorphic tumor cells along with areas showing nuclear overlapping and overcrowding. Some tumor cells displayed optically clear chromatin, nuclear grooving, and nuclear pseudoinclusion (Figure 1C). The mesenchymal element appeared mild to moderately cellular, and contained uniform, spindle-shaped stromal cells in fascicular arrangement (Figure 1D).

**Figure 2:**
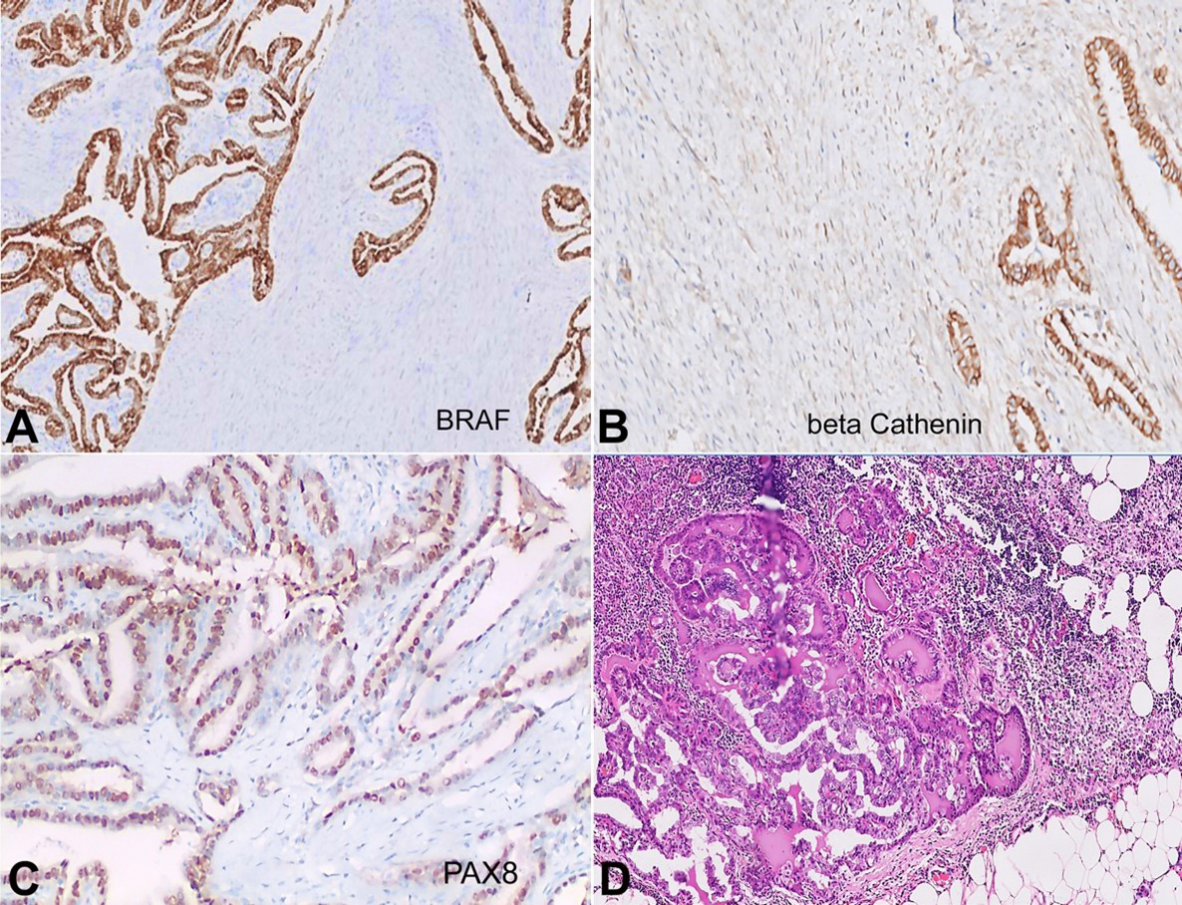
**A** – The epithelial component shows diffuse strong cytoplasmic granular positivity for the BRAF mutant protein (10x); **B** – diffuse weak membranous positivity for β-catenin (20x); **C** – and variable nuclear positivity for PAX8 (20x). The stromal component was negative for BRAF, β-catenin and PAX8 immunostains. **D** – Lymph node metastasis showing only the epithelial component (H&E, 20x).

No significant atypia, mitotic activity, or necrosis was evident within the stroma. Two nodules in the isthmus region displayed features of nodular hyperplasia; no evidence of papillary carcinoma was noted. On immunohistochemistry (IHC) assessment (Table 1), the epithelial component showed expression for TTF-1, thyroglobulin, *BRAF* V600E mutant protein (VE1 clone, Ventana) (Figure 2A), beta-catenin (membranous positivity; Figure 2B), PAX8 (Figure 2C), pan cytokeratin, CK7, and BCL2 (weak). The stromal cells showed positivity for smooth muscle actin (focal) and vimentin, whereas it was negative for beta-catenin and BCL2. Desmin, CK19, CD31, CD34, CD117, p53, and S100p, all were negative in both the components. Ki-67 proliferating index was <5% and <2% in the epithelial and stromal components, respectively. DNA was extracted from formalin-fixed paraffin-embedded tissue and was subjected to the *BRAF* gene (exon 15) sequencing, which revealed the *BRAF*V600E (c.1799T>A, Val600Glu) mutation. Two lymph nodes in the central quadrant showed evidence of metastasis. However, in the metastatic site, only the epithelial component was detected, showing features of classical papillary carcinoma without the presence of a stromal component (Figure 2D). Based on these features, a diagnosis of PTC-NFS, pT2N1aMx (*AJCC Cancer Staging Manual, Eighth Edition*) was made.^2^

**Table 1:** Immunohistochemistry findings in the index case

IHC markers	PTC component	STROMAL component	IHC markers	PTC component	STROMAL component
BRAF V600E	+	-	SMA	-	+ (focal)
Beta-catenin	+	-	Desmin	-	-
TTF-1	+	-	CD31	-	-
Thyroglobulin	+	-	CD34	-	-
Pan-Cytokeratin	+	-	CD117	-	-
CK7	+	-	BCL2	±	-
CK19	-	-	S100+	-	-
PAX8	+	-	Vimentin	-	+
P53	-	-	Ki-67	<5%	<2%

IHC = immunohistochemistry; PTC = papillary thyroid carcinoma; TTF-1 = thyroid transcription factor -1; CK = cytokeratin; SMA = smooth muscle actin; + = positive; - = negative.

Postoperatively, two USG scans (3 months apart) were performed, which did not reveal any residual lesion. Stimulated serum thyroglobulin (4.06 ng/mL) and anti-thyroglobulin (106 IU/mL) were also within the normal range. Subsequently, the low-dose whole-body radioiodine scan performed was also negative. The patient was doing well at follow-up of 9 months.

## DISCUSSION

Many histological variants of PTC are recognized and most of them carry an excellent prognosis owing to their indolent biological behavior. The recent 2017 edition of *WHO Classification of Tumors of Endocrine Organs* 1 mentions PTC with fibromatosis/fasciitis-like stroma as an extremely rare variant, although Ostrowski et al.^3^ and Chan et al.^4^ were the first to described it as PTC-DTF and PTC-NFS, respectively. There are no specific cut-off criteria for each component to qualify; in the majority, the proliferating stroma significantly surpasses the epithelial component. In the English literature, there are approximately 30 cases reported in the form of case reports and case series, and to the best of our knowledge, very few of them are from the Indian population.^5^^-^ 11 It has been reported over a wide age range (19–77 years) without any gender predilection. Our index case exhibited a favorable clinical course with no residual disease as at the last follow-up.

PTC-DTF is differentiated from PTC-NFS by β-catenin nuclear immunoexpression in the stromal component as well as activating *CTNNB1* mutations.^12^ A detailed molecular work-up established that *BRAF* and *CTNNB1* mutations were restricted to the epithelial and stromal components, respectively.^9^^,^ 10 The *CTNNB1* gene encodes for β-catenin that drives the Wnt/β-catenin signaling pathway for cell growth and/or survival. The cases, without β-catenin expression or *CTNNB1* mutation, were considered as PTC-NFS, which is in concordance with our index case. However, both PTC-DTF and PTC-NFS follow a generally favorable course when managed in a similar way to conventional papillary thyroid carcinoma.^13^ The *MYH9-USP6* rearrangement is commonly seen in 90% of soft tissue nodular fasciitis; however, its presence has not been established in PTC-NFS.^7^ Most of the previous studies examining *BRAF* mutation in PTC-DTF found that the activation of the *BRAF* Val600Glu mutation is the most common genetic alteration seen in the epithelial component.^7^^,^ 10 Information on PTC-NFS is extremely limited. The exact pathogenesis behind the stromal cell proliferation in PTC-NFS, whether reactive or neoplastic, still remains cryptic and needs to be determined in the future. Our index case showed the *BRAF* V600E mutation. IHC showed *BRAF* mutant protein expression only in the epithelial component, indicating that the epithelial component is neoplastic while the stromal component is likely reactive. Interestingly, in the index case at the metastatic site, the tumor showed only the epithelial component. This further indicates that the stromal component is probably reactive. However, we did not perform a *CTNNB1* gene mutation or next generation sequencing. In the English literature, approximately 50% of cases reported so far showed lymph node metastasis. Although the number of reported cases is small and there is insufficient follow-up in most of the reports, it is suggested that the PTC/NFS and/or PTC/DTF seems to carry a similar prognosis to conventional PTC, irrespective of the lymph node status.

FNA is commonly used for the diagnosis of thyroid neoplasms. The presence of only the stromal component makes the diagnosis of PTC-NFS more difficult and challenging in FNA. A literature search revealed that FNA cytology was used to diagnose PTC-NFS or PTC-DTF in 13 cases, out of which both epithelial and stromal components were identified (7 cases), while in 5 cases, only the epithelial component was identified. However, in 1 case, the authors identified only the stromal component, which was misdiagnosed as a sarcoma on FNA.^14^ The differential diagnoses in such case could be benign or malignant spindle cell tumors. However, most of them can be excluded on the basis of the clinical, radiological, histopathological, and appropriate immunohistochemical findings. The worrisome differential for the clinician as well as the pathologists is the aggressive anaplastic thyroid carcinoma, which is known to show spindle-shaped cells in sarcoma-like fascicles. These can be distinguished as most of them display keratin immunoreactivity.^1^ In the present case, the above differentials were considered and ruled out.

## CONCLUSION

PTC-NFS/DTF is a histological variant of PTC with a favorable prognosis. Our index case was associated with the *BRAF* mutation, which was restricted to the epithelial component. Thorough sampling of the excised specimen is essential in order not to miss the epithelial component, which, in most reported cases—including ours—appears to be small.
